# Genome-Wide Analysis of Cotton Auxin Early Response Gene Families and Their Roles in Somatic Embryogenesis

**DOI:** 10.3390/genes10100730

**Published:** 2019-09-20

**Authors:** Ruibin Sun, Shaohui Wang, Dan Ma, Yilin Li, Chuanliang Liu

**Affiliations:** 1 State Key Laboratory of Cotton Biology, Institute of Cotton Research, Chinese Academy of Agricultural Sciences, Anyang 455000, China; 2 Zhengzhou Research Base, State Key Laboratory of Cotton Biology, Zhengzhou University, Zhengzhou 450001, China

**Keywords:** *Gossypium hirsutum*, somatic embryogenesis, *ARF*, *Aux/IAA*, *GH3*, *SAUR*

## Abstract

Auxin is well known to regulate growth and development processes. Auxin early response genes serve as a critical component of auxin signaling and mediate auxin regulation of diverse physiological processes. In the present study, a genome-wide identification and comprehensive analysis of auxin early response genes were conducted in upland cotton. A total of 71 *auxin response factor* (*ARF*), 86 *Auxin/Indole-3-Acetic Acid* (*Aux/IAA*), 63 *Gretchen Hagen3* (*GH3*), and 194 *small auxin upregulated RNA* (*SAUR*) genes were identified in upland cotton, respectively. Phylogenetic analysis revealed that the *ARF*, *GH3*, and *SAUR* families were likely subject to extensive evolutionary divergence between *Arabidopsis* and upland cotton, while the *Aux/IAA* family was evolutionary conserved. Expression profiles showed that the *ARF*, *Aux/IAA*, *GH3*, and *SAUR* family genes were extensively involved in embryogenic competence acquisition of upland cotton callus. The *Aux/IAA* family genes generally showed a higher expression level in the non-embryogenic callus (NEC) of highly embryogenic cultivar CCRI24 than that of recalcitrant cultivar CCRI12, which may be conducive to initializing the embryogenic transformation. Auxin early response genes were tightly co-expressed with most of the known somatic embryogenesis (SE) related genes, indicating that these genes may regulate upland cotton SE by interacting with auxin early response genes.

## 1. Introduction

Upland cotton (*Gossypium hirsutum*) is an important economic crop, produces large amounts of natural fibers as raw material for the textile industry, and is used to extract nutrient-rich edible oil from cottonseed [1,2]. Given its substantial economic worth, the improvement of yield, fiber quality, stress tolerance, disease resistance, and other desired traits of cotton are important. Genetic improvement of cotton by transgenesis or genome editing technology is a mainstay for cotton breeding to widen the germplasm genetic diversity and generate transgenic cotton varieties with improved agronomic traits [3]. In cotton, *Agrobacterium*-mediated genetic transformation has been the most widely used approach to incorporate exogenous genes into explants [4,5]. However, this transformation method is genotype-dependent, and upland cotton with different genotypes has a variable somatic embryogenesis ability during regeneration. Although a relatively effective regeneration system has been established in several upland cotton cultivars, most of the desirable cultivars with elite traits remain difficult to regenerate [4,6,7]. The inability of regeneration via somatic embryogenesis severely constrains cotton genetic engineering.

Somatic embryogenesis is a complicated multi-step process whereby the transformed explant cells/tissues dedifferentiate into callus, then acquire the embryogenic potency to develop into a somatic embryo, which ultimately grows to be a plantlet [5,8]. The acquirement of embryogenic potential, viz the transition from non-embryogenic callus to embryogenic callus, is very crucial for somatic embryogenesis and has been suggested as the key bottleneck step for the regeneration of cotton [9,10]. Phytohormones have been found to be extensively involved in somatic embryogenesis in many plant species [11], of which auxin and cytokinin have been suggested to be the most important factors affecting the acquisition of embryogenic potential. In cotton, endogenous auxin was significantly increased in embryogenic callus when compared to non-embryogenic callus, and large numbers of auxin signal transduction related transcripts were identified during the transformation from non-embryogenic callus (NEC) to embryogenic callus (EC) [12,13,14]. This suggests that auxin signaling and response might play important roles in acquiring the embryogenic competence of cotton callus.

*Auxin response factors* (*ARFs*), *Auxin/Indole-3-Acetic Acids* (*Aux/IAAs*), *Gretchen Hagen3s* (*GH3s*), and *small auxin upregulated RNAs* (*SAURs*) have been identified as major early auxin responsive genes, serve as critical component of auxin signaling and homeostasis, regulate downstream auxin late response genes in the transcriptional network, and further mediate auxin regulated diverse physiological processes [15,16,17]. *ARF* genes encode a class of auxin response transcription factors, which contains several described functional domains: a plant specific B3-type DNA binding domain (DBD) at the amino-terminal, a transcriptional activation or repression domain in the middle region (MR), and a carbon-terminal dimerization domain (CTD) in most cases [18]. DBD makes the ARFs specifically bind to the auxin response cis-element (*AuxRE*) in the promoters of auxin response genes such as *GH3s* and *SAURs*. The MR domain functions to activate or repress the expression of targeted genes when ARFs are recruited by *AuxRE*. CTD enables ARFs to dimerize with other ARFs or interact with heterologous co-regulation factors such as Aux/IAA [16,18]. The *Aux/IAA* family genes encode short-lived transcriptional regulator proteins that contain four conserved domains referred to as domain I to IV. The domain III and domain IV sequences are highly homologous with the CTD regions of ARFs and contribute to the interaction of Aux/IAA and ARF [19]. In auxin regulated gene expression, Aux/IAA acts as a repressor of the transcription of auxin response genes by recruiting associated corepressors to interact with ARF, resulting in the repression of the activation activity of ARF [20]. While Aux/IAA protein homeostasis is auxin dependent, Aux/IAA can interact with the auxin receptor protein TRANSPORT INHIBITOR RESPONSE1 (TIR1) and form the TIR1-Aux/IAA complex. When high levels of auxin are perceived by TIR1, the formation of the auxin-TIR1-Aux/IAA complex triggers the degradation of the Aux/IAA repressor, which derepressed the activity of ARF and enabled the activation of targeted auxin response genes [16,21]. Thus, the Aux/IAA interacted CTD of the ARF confers the expression regulation of auxin inducibility of *ARFs*. Through the mechanism based on Aux/IAA-ARF interaction, ARFs and Aux/IAAs play important roles in regulating the expression of diverse auxin response genes. The *GH3* family genes participate in the maintenance of auxin, SA, and JA homeostasis by conjugating excess indole-3-acetic acid to amino acids [16,22], and serves as an essential constituent of the auxin regulation network. *SAUR* genes comprise a large multigene family that encode small auxin-induced RNAs, and the transcription of *SAUR* genes can be significantly and sharply induced within 2–5 minutes during auxin signaling [23,24]. SAUR proteins act as key effectors of hormone signals in regulating plant growth and development [25]. In cotton somatic embryogenesis, auxin plays a critical role in a complex manner. *ARF* and *Aux/IAA* genes were found differentially expressed during the initial differentiation of somatic embryogenesis in different upland cotton cultivars [26]. Endogenous indoleacetic acid (IAA) content fluctuates in different stages of somatic embryogenesis and surges in embryogenic callus, followed by numerous auxin responsive genes including *ARF*, *Aux/IAA*, *GH3*, and *SAUR* genes differentially expressed during this developmental process [13,27]. Similar results were observed in other studies. Xu et al. (2013) found that endogenous IAA concentration significantly increased during embryogenic competence acquisition in upland cotton somatic embryogenesis, with a number of auxin biosynthesis and signal transduction related genes differentially expressed between non-embryogenic callus and embryogenic callus [12]. Furthermore, auxin early response genes including *ARF*, *Aux/IAA*, *GH3*, and *SAUR* family members showed significant differential expression between sibling cotton lines with distinct different somatic embryogenesis ability, with some auxin response genes even showing specific expression in a highly embryogenic line [12]. All of these suggest the underlying important roles of auxin response genes in somatic embryogenesis.

As previous studies have indicated, auxin response genes are closely implicated in the acquisition of embryogenic competence in cotton somatic embryogenesis [12,28]. We conducted a genome-wide identification and comprehensive analysis of auxin early response genes in cotton, and explored their regulatory roles and mechanisms in the key step of embryogenic competence acquisition in cotton somatic embryogenesis. Our study provides valuable insights into the auxin signaling profile and regulatory roles in the cotton somatic embryogenesis.

## 2. Materials and Methods

### 2.1. Identification of ARF, Aux/IAA, GH3, and SAUR Genes in Upland Cotton

Newly assembled genomic data and gene annotation of *G. hirsutum* was downloaded from public website (http://cotton.hzau.edu.cn/EN/download.php) as described in the corresponding published paper [29]. To identify the gene family members, the hidden Markov models (HMMs) of *ARF* (PF06507), *Aux/IAA* (PF02309), *GH3* (PF03321), and *SAUR* (PF02519) were downloaded from the Pfam database (http://pfam.xfam.org/) [30], which then served as queries for the homologues search (*E-value* < 1) against the proteome of *G. hirsutum* conducted by HMMER v.3.1b2 software [31]. In addition, we performed a similarity search (*E-value* < 1 × 10^−5^, identity > 50%) against the proteome of *G. hirsutum* by BLAST+ v.2.6.0 [32] with the amino acid sequences of previously reported *Arabidopsis* ARFs, Aux/IAAs, GH3s, and SAURs as queries. The sum total resulting protein hits from the HMMER search and BLAST search were used for subsequent filtering. Furthermore, InterProScan v.5.32-71.0 [33] was used as the domain prediction to test the presence of characteristic domains. Items containing “Auxin response factor” domain (IPR010525) were recognized as ARFs; items containing the “Aux/IAA” domain (IPR033389) or “PB1” domain (IPR000270), but no “Auxin response factor” domain were recognized as Aux/IAAs; items containing the “GH3” domain (IPR004993) were recognized as GH3s; and items containing the “Small auxin-up RNA” domain (IPR003676) were recognized as SAURs.

### 2.2. Phylogenetic Analysis and Gene Structure of Upland Cotton ARF, Aux/IAA, GH3, and SAUR Genes

Amino acid sequences of *ARF*, *Aux/IAA*, *GH3*, and *SAUR* family genes identified in *G. hirsutum* and previous reported *Arabidopsis* members were combined to perform multiple sequences alignment by MUSCLE [34]. The phylogenetic tree was constructed by IQTREE [35] using the maximum likelihood (ML) statistical method and the best substitution model selected automatically.

Coordinates of the exon–intron and characteristic domains of *ARF*, *Aux/IAA*, *GH3*, *SAUR* genes were extracted from the genomic gene annotation and InterProScan annotation results, respectively. TBtools was used to display the gene structure and characteristic domain coding regions.

### 2.3. Genomic Distribution, Collinearity and Duplication Analysis of Upland Cotton ARF, Aux/IAA, GH3, and SAUR Genes

Based on the genomic coordinates of the *G. hirsutum ARF*, *Aux/IAA*, *GH3*, and *SAUR* genes retrieved from genomic gene annotation, the R package RIdeogram [36] was used to map genes on the chromosomes. MCScanX [37] was used to identify collinearity blocks in *G. hirsutum*. Collinearity relations of *ARF*, *Aux/IAA*, *GH3*, and *SAUR* genes were displayed by the circular figure plotted by Circos v.0.69 [38]. Genes in the same family were recognized as derived from segmental duplication when located in collinear blocks. Tandem duplications were detected using MCScanX with the default parameters.

### 2.4. Expression Profile Analysis of ARF, Aux/IAA, GH3, and SAUR Genes during Embryogenic Ability Acquisition of Upland Cotton Callus

We previously conducted RNA sequencing of the key step of cotton somatic embryogenesis, where non-embryogenic callus (NEC) and embryogenic callus (EC) from two upland cotton cultivars with different somatic embryogenesis ability (recalcitrant cultivar CCRI12 and highly embryogenic cultivar CCRI24) were sampled (NEC and EC forming CCRI12 are referred to as AN and AE, respectively, NEC and EC forming CCRI24 are referred as BN and BE, respectively) and sequenced using the Illumina platform [28]. This enabled us to investigate the expression profiles of auxin early response genes during cotton somatic embryogenesis. By mapping the sequencing reads to the newly assembled *G. hirsutum* genome as a reference using Hisat 2 [39], StringTie [40] was used to conduct the quantification of genes and transcripts. Transcripts per million (TPM) read values were computed to represent the gene expression levels. Heatmaps with hierarchy clustering were plotted based on log 10-transformed TPM values using the R software package pheatmap to visualize the gene expression profiles.

### 2.5. Differentially Expression Analysis

We identified differentially expressed genes (DEGs) for a comparison of different tissue types and cultivars. DESeq 2 [41] was used for different ially expressed gene (DEG) identification based on the read count matrix derived from the StringTie results. Genes showing an absolute value of expression level fold change (FC, represents the ratio of expression level as the TPM value between different samples in a comparison pair) > 2 and q-value < 0.05 were recognized as DEGs. Over-representation analysis by the hypergeometric test method was conducted using the R package clusterProfiler v.3.6.0 [42] based on all of the identified DEGs. To avoid the defects of a hypergeometric test that only focus on genes with a large fold change, the gene set enrichment analysis (GSEA) was conducted based on all expressed genes. Each *ARF*, *Aux/IAA*, *GH3*, and *SAUR* gene family was defined as a gene set. The *p*-value resulting from DESeq 2 was preprocessed to be transformed into a metric for each gene. Resulted metrics were used as the input of GSEA by running the GSEAPreanked tools in the Broad Institute’s GSEA software [43].

To further compare the expression patterns of differentially expressed *ARF*, *Aux/IAA*, *GH3*, and *SAUR* genes during callus embryogenic potential acquisition, heatmaps were plotted based on the log 2-transformed gene TPM values.

### 2.6. Co-Expression Network Analysis

Based on the expression profiles of all the differentially expressed genes during upland cotton somatic embryogenesis, we computed the Pearson’s correlation coefficients between gene pairs using R. Genes with correlation coefficients > 0.98 were recognized as co-expressed. Co-expression between auxin early response genes and somatic embryogenesis related genes were focused and a co-expression network was constructed and displayed by Cytoscape v.3.7.1 [44].

### 2.7. qRT-PCR Validation and Analysis

qRT-PCR was conducted to validate the expression profiles of auxin early response genes derived from RNA sequencing. Twelve representative genes including two *ARF*, six *Aux/IAA*, two *GH3*, and two *SAUR* genes were selected for qRT-PCR validation. Specific primers were designed using the National Center for Biotechnology Information (NCBI) Primer-BLAST (https://www.ncbi.nlm.nih.gov/tools/primer-blast/index.cgi) and synthesized commercially (Sangon Biotech). cDNA was synthesized from RNA samples of NEC and EC of CCRI12 and CCRI24 using a HiScript III 1st Strand cDNA Synthesis Kit (Vazyme Biotech Co. Ltd, Nanjing, China) according to the manufacturer’s instructions. The obtained cDNA products were diluted five times prior to amplification. qPCR was conducted with a 20 μL reaction system containing 1 μL cDNA templates, 10 μL 2 × TransStart Top Green qPCR Super Mix (TransGen Biotech Co. Ltd, Beijing, China), 0.5 μL of each 10 μM forward and reverse primers, and 8 μL ddH_2_O. All qPCR reactions were performed in triplicate on a Roche Light Cycler 480 instrument. A thermal cycling program was performed with pre-incubation at 95 °C for 5 min, followed by 40 cycles of denaturation at 95 °C for 10 s, annealing at 58 °C for 10 s and extension at 72 °C for 10 s. Relative quantitation of gene expression was computed using the online tool A shiny for the analysis of real-time PCR data (https://ihope.shinyapps.io/qRT-PCR-Pipeline/) with *GhHis3* (GenBank: AF024716) was used as the internal reference. 

## 3. Results

### 3.1. Identification and Characterization of ARF, Aux/IAA, GH3, and SAUR Genes in Upland Cotton

A total of 71 *ARF*, 86 *Aux/IAA*, 63 *GH3*, and 194 *SAUR* genes were identified in upland cotton. All of the identified upland cotton ARFs have a B3-type DBD, ARF domain, and Aux/IAA-like CTD at the same time. Of these, 24 ARFs whose MR were enriched in glutamine (Q), serine (S), and leucine (L) were considered as activators, while other ARFs rich in serine (S), proline (P), and leucine (L) were probable transcriptional repressors [18,45] (Table 1, Supplementary Figure S1). Most of the identified upland cotton Aux/IAAs harbored all of the typical four conserved domains (Domains I–IV), while some of the Aux/IAAs in upland cotton were truncated. Sixteen Aux/IAAs lacked the “LxLxL” motif-containing Domain I, which has the function of ethylene response factor-associated amphiphilic repression. Two Aux/IAAs lacked Domains I and II at the same time. Two and three Aux/IAAs lacked Domains III and IV, respectively, one Aux/IAA lacked Domains III and IV at the same time, while the other lacked Domains III and II at the same time (Table 1). The canonical degron motif “GWPPV”, which confers rapid auxin-induced Aux/IAA degradation, existed in the Domain II of upland cotton Aux/IAAs [19,46]. Additionally, the previously reported rate motif “KR” located between Domains I and II is present in upland cotton Aux/IAAs and controls the auxin-induced degradation rate of Aux/IAAs [47] (Supplementary Figure S2). All identified upland cotton GH3s had a conserved GH3 domain; within the eight GH3s contained 2 × GH3 domains, while the other eight GH3s contained the Jasmonic acid-amino synthetase (JAR1) domain simultaneously (Table 1). Previous study showed that some conserved motifs/residues are necessary for GH3s’ hormone-amino synthetase activity including four hormone-binding motifs/residues and three ATP/AMP-binding motifs/residues [48]. In the present study, almost all identified upland cotton GH3s contained all of these functional motifs/residues, except for three C-terminal truncated GH3s (Ghir_A04G003360.1, Ghir_A01G005350.1, Ghir_A01G008830.1) and two N-terminal truncated GH3s (Ghir_D04G015220.1, Ghir_A11G031180.1) lacking some of functional motifs/residues, and in particular, a severely truncated GH3 (Ghir_A11G031160.1) contained only one ATP/AMP-binding motif/residue (Supplementary Figure S3). All identified upland cotton SAURs contained a SAUR domain and auxin-inducible motif (Table 1, Supplementary Figure S4). The identification results of the auxin early response genes in the present study was almost consistent with previous studies. The number of *G. hirsutum ARF* genes (71) identified in the present study was almost the sum of the number of *ARF* genes in its diploid ancestor species as each *G. arboreum* and *G. raimondii* contained 35 *ARF* genes [49,50]. Previous study identified 56 *GH3* genes in *G. hirsutum* (NAU-NBI, v1.1) [48,51], while 63 were identified in the present study. Nevertheless, the present study identified the same number of *GH3* subfamily II gene members as the previous study [48]. The present study identified fewer *SAUR* genes than the previous study [52]. In a previous study, more than twenty of the identified *SAUR* genes were located on scaffolds, while all 194 *SAUR* genes identified in the present study were located on chromosomes [52]. The difference in the identification results may be due to the different genome assembly and identification methods adopted. We supposed that some scaffolds may be assembled onto chromosomes in the new genome assembly versus the one before, making a more accurate identification of the gene members.

### 3.2. Phylogenetic Analysis and Gene Structure of ARF, Aux/IAA, GH3, and SAUR Genes in Upland Cotton

**Figure 1:**
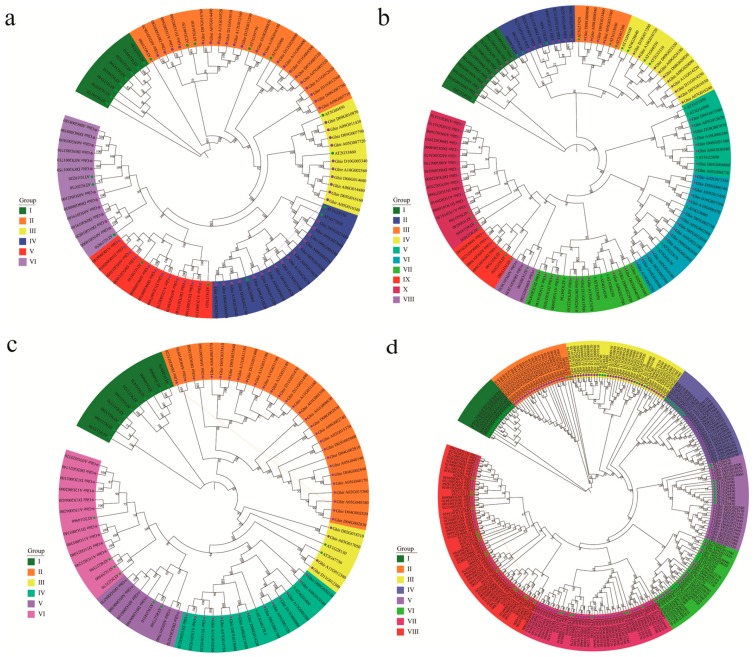
Phylogenetic trees of *ARF* (**a**), *Aux/IAA* (**b**), *GH3* (**c**), and *SAUR* (**d**) family genes from *Arabidopsis* and upland cotton. The trees were constructed by the maximum likelihood (ML) method based on the full length amino acid sequence of the ARF, Aux/IAA, GH3, and SAUR proteins.

We constructed phylogenetic trees by the maximum likelihood (ML) method based on full length amino acid sequences of identified ARF, Aux/IAA, GH3, and SAUR protein in upland cotton with corresponding gene family members in *Arabidopsis* as a reference (Figure 1). Results showed that most clades had robust bootstrap support, and the topological classification of *Arabidopsis* genes in our ML trees were consistent with previous studies. According to the ML tree, *ARF* genes were classified into six distinct groups. Group I contained genes only from *Arabidopsis*, while all of the other groups were shared by *Arabidopsis* and upland cotton genes (Figure 1a). All transcriptional activators of ARFs were distributed in Groups V and VI. The *Aux/IAA* genes were classified into ten groups, all groups were shared by *Arabidopsis* and upland cotton (Figure 1b). *GH3* genes were divided into six groups, except for Group I where all members belonged to *Arabidopsis,* and Group II where all members belonged to upland cotton; all other groups were shared with *Arabidopsis* and upland cotton (Figure 1c). The *SAUR* genes were divided into eight groups, almost all groups were shared by *Arabidopsis* and upland cotton, except for Group I, which consisted of only *Arabidopsis* gene members (Figure 1d). These results suggest that the *Aux/IAA* family genes are relatively conserved between *Arabidopsis* and upland cotton during evolution, while the *ARF*, *GH3*, and *SAUR* family genes may experience more extensive evolutionary divergence as some clades exhibit species specificity.

Gene exon–intron structure analysis showed that genes in each clade exhibited comparable exon–intron structure between *Arabidopsis* and upland cotton (Supplementary Figure S5), implying the evolutionary conservation of these gene members between *Arabidopsis* and upland cotton. Gene exon–intron structure and intron number maintained relatively stable within groups, while it varied between different groups in the *ARF*, *Aux/IAA*, and *GH3* families, which almost coincided with the classification in the ML trees. The *ARF* genes had the most introns (average 10.38 ± 4.35), while the *Aux/IAA* and *GH3* genes had less introns (average 4.52 ± 1.10 and 3.90 ± 0.86, respectively). Interestingly, the majority of upland cotton *ARF* genes had 10 to 15 introns, while the *ARF* gene members in Group IV contained significantly less introns, which varied from 2 to 4. The majority of *SAUR* genes had no introns, and this could be attributed to the short length of *SAUR* genes (most *SAUR* genes are less than 1 kb).

### 3.3. Genomic Distribution and Gene Duplication of ARF, Aux/IAA, GH3, and SAUR Genes in Upland Cotton

**Figure 2:**
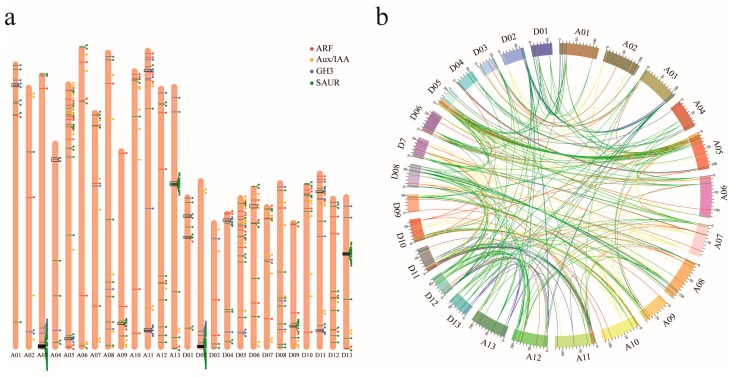
Chromosomal distribution (**a**) and collinearity (**b**) of the identified *ARF*, *Aux/IAA*, *GH3*, and *SAUR* genes in upland cotton indicated gene duplications. (**a**) Genes were displayed by striping on chromosomes, circle points were labeled at the end of stripes to make a clearer distinction between adjacent genes from each other. *ARF*, *Aux/IAA*, *GH3*, and *SAUR* genes are represented by red, yellow, blue, green stripes and labels, respectively. Tandem duplicate genes were surrounded by black rectangles to represent tandem duplication gene sets. (**b**) Chromosomal segmental duplication regions were analyzed by MCScanX, *ARF*, *Aux/IAA*, *GH3*, and *SAUR* homologous gene pairs in segmental duplication regions linked with different color curves (red, yellow, blue, and green, respectively).

Based on the genes coordinate annotation data, we mapped identified upland cotton *ARF*, *Aux/IAA*, *GH3*, and *SAUR* genes on chromosomes (Figure 2a). All of these genes are located on assembled chromosomes and are distributed unevenly across different chromosomes, with none on the scaffolds. All 71 identified *ARF* genes were distributed on 24 of the total 26 chromosomes; no *ARF* gene was located on chromosomes A04 and D02. Similarly, 86 *Aux/IAA* genes were distributed on 21 chromosomes; moreover, there was no *Aux/IAA* gene on chromosomes A01, A03, A04, D01, and D04. Moreover, 63 *GH3* genes were distributed on 20 chromosomes, with no *GH3* gene on chromosomes A06, A09, A10, D06, D09, and D10. Additionally, 194 *SAUR* genes were distributed across all 26 upland cotton chromosomes with dense distributions on the terminal of chromosomes A03 and D02, which was largely similar to a previous study [52]. Based on the results of the collinearity analysis, we found that the majority of *ARF*, *Aux/IAA*, *GH3*, and *SAUR* genes belonged to segmental duplicates resulting from polyploidy and chromosome rearrangements. There were 59 *ARF*, 90 *Aux/IAA*, 38 *GH3*, and 187 *SAUR* homologous gene pairs involving 71 *ARF* genes, 85 *Aux/IAA* genes, 55 *GH3* genes, and 175 *SAUR* genes located in segmental duplication blocks, respectively (Figure 2b). Furthermore, one, 10, and 17 gene sets involving two *Aux/IAA* genes, 23 *GH3* genes, and 43 *SAUR* genes, respectively, were determined as tandem duplication, and no tandem duplication was observed in upland cotton *ARF* genes (Figure 2a). This implied that segmental duplication extensively existed in the *ARF*, *Aux/IAA*, *GH3*, and *SAUR* gene families, and tandem duplication further gave rise to more gene members of the *GH3* and *SAUR* families.

### 3.4. Expression Profiling and Differentially Expression of ARF, Aux/IAA, GH3, and SAUR Genes in Upland Cotton Somatic Embryogenesis

**Figure 3:**
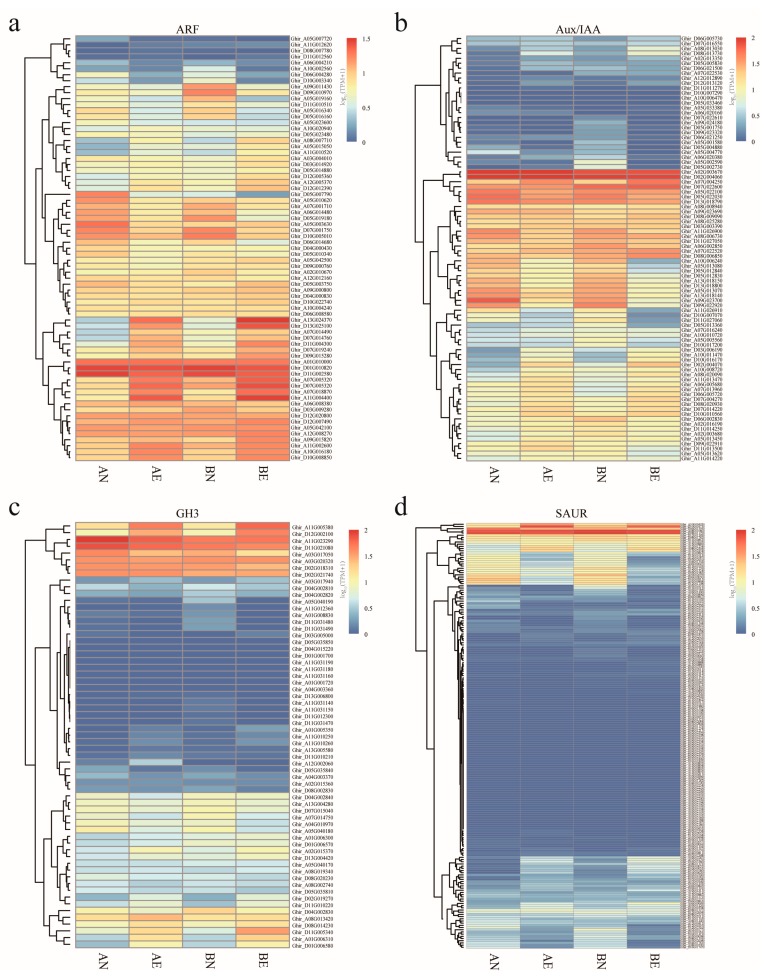
Expression profiles of *ARF* (**a**), *Aux/IAA* (**b**), *GH3* (**c**), and *SAUR* (**d**) genes during the acquisition of the embryogenic ability of upland cotton callus from NEC to EC. The heatmaps were drawn based on log 10-transformed TPM values of genes derived from the RNA sequencing analysis. AN and AE represent the NEC and EC of the recalcitrant embryogenic cultivar CCRI12, respectively and BN and BE represent the NEC and EC of highly embryogenic cultivar CCRI24, respectively.

As the auxin signaling pathway was found to play an important role in cotton somatic embryogenesis, we focused on the expression pattern of auxin early response *ARF*, *Aux/IAA*, *GH3*, and *SAUR* genes during this process. We previously conducted RNA sequencing on the bottleneck step of upland cotton somatic embryogenesis and the acquisition of embryogenic potential in the transformation from non-embryogenic callus (NEC) to embryogenic callus (EC) [28] by using two upland cotton cultivars with different somatic embryogenesis ability (recalcitrant embryogenic cultivar CCRI12 and highly embryogenic cultivar CCRI24). This enabled us to elucidate and compare the transcriptomic dynamics of *ARF*, *Aux/IAA*, *GH3*, and *SAUR* genes in different cultivars. As a result, the majority of *ARF* and *Aux/IAA* genes were actively expressed during the acquisition of embryogenic ability. About 94.4% (67 out of 71) of *ARF* genes and 83.7% (72 out of 86) of *Aux/IAA* genes were expressed (TPM value > 1) during the embryogenic transformation of the callus, and 90.1% (64 out of 71) of *ARF* genes and 66.3% (57 out of 86) of *Aux/IAA* genes were expressed at relatively high levels with TPM > 5. Almost half of the *GH3* genes (37 out of 63) showed expression (TPM value > 1) in this process, with 27 *GH3s* showing a TPM > 5. While the majority of *SAUR* genes showed a low level of expression throughout the embryogenic ability acquisition with only 18.0% (35 out of 194) of *SAUR* genes actively expressed with a TPM value > 5 (Figure 3, Table S1). This suggests that *ARF* and *Aux/IAA* family genes extensively participate in the auxin signaling mediated embryogenic ability acquisition process during upland cotton somatic embryogenesis, where some of the *GH3* family gene members were also involved in this process, while only a few *SAUR* genes were involved.

**Figure 4:**
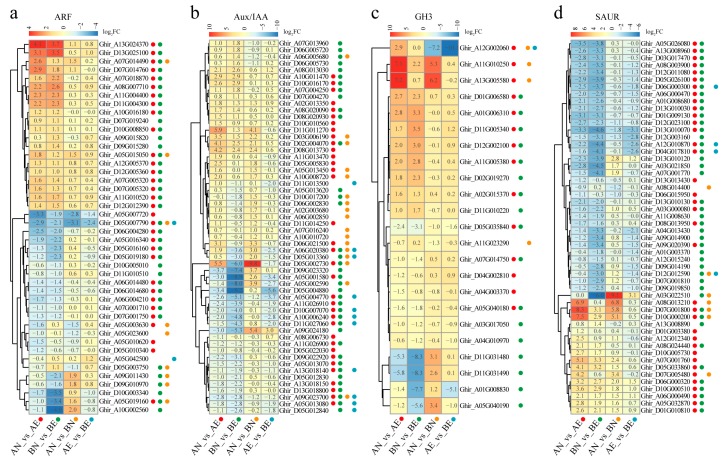
Schematic expression level change of differentially expressed *ARF* (**a**), *Aux/IAA* (**b**), *GH3* (**c**), and *SAUR* (**d**) genes during transformation from NEC to EC. Numbers in grids indicate the log 2-transformed fold change (FC) values. Colored circle points beside the gene names indicate if the corresponding genes were significantly differentially expressed in AN_vs_AE (red), BN_vs_BE (green), AN_vs_BN (orange), and AE_vs_BE (blue) comparison pairs.

**Figure 5:**
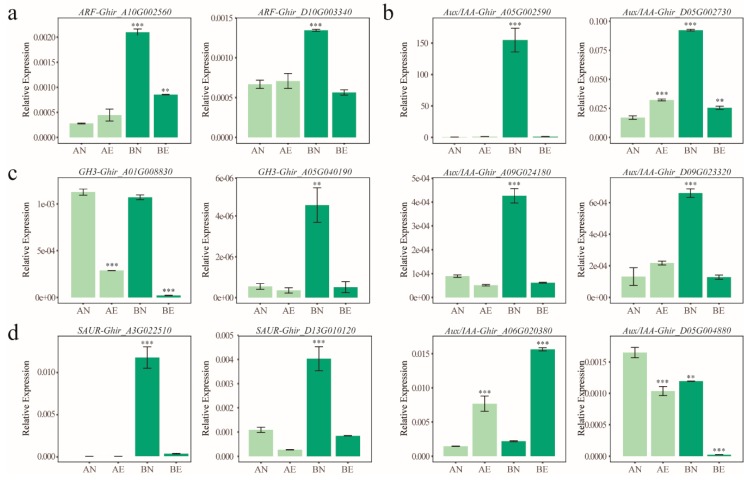
qRT-PCR analysis showing the expression of auxin early response *ARF* (**a**), *Aux/IAA* (**b**), *GH3* (**c**), and *SAUR* (**d**) genes during transformation from NEC to EC. Callus samples from CCRI12 and CCRI24 were colored in light green and dark green, respectively. Multiple comparisons of expression levels between different samples were conducted with AN chosen as the reference. Statistically significant differences are displayed with asterisks (‘***’ *p*-value ≤ 0.001, ‘**’ *p*-value ≤ 0.01).

Further analysis found that there were 44 *ARF* (62.0%), 56 *Aux/IAA* (65.1%), 23 *GH3* (36.5%), and 52 *SAUR* (26.8%) genes that were significantly differentially expressed during the process from NEC to EC when using an absolute value of fold change (FC, represents the ratio of expression level as TPM value between different samples in a comparison pair) > 2 and *q*-value < 0.05 as the criteria (Figure 4, Table 2). For all four auxin early response gene families, comparisons of AN_vs_AE and BN_vs_BE made up the majority of these differentially expressed genes (DEGs), while few auxin early response genes showed significant differentially expression in comparisons AN_vs_BN and AE_vs_BE (Table 2). Twelve differentially expressed auxin early response genes were selected to validate the expression pattern through qRT-PCR (Table S2). The results showed that almost all tested genes showed comparable expression patterns between the qRT-PCR measurements and RNA sequencing analysis, except for one *Aux/IAA* gene (Figure 5). The qRT-PCR validation results suggest the reliability of RNA sequencing.

In most cases, auxin early response genes showed the same expression pattern between the AN_vs_AE and BN_vs_BE comparisons, suggesting that this part of the gene may be critical for callus embryogenic ability acquisition in both the CCRI12 and CCRI24 cultivars (Figure 4). With some exceptions, the *Aux/IAA* gene *Ghir_D05G002730* showed the opposite expression pattern between AN_vs_AE and BN_vs_BE, which was significantly upregulated in CCRI12, but significantly downregulated in CCRI24 during the process from NEC to EC (Figure 4 and Figure 5), implying the transcriptional response difference between different cultivars during embryogenic ability acquisition, which may be implicated in the difference of somatic embryogenesis ability. Moreover, concerning the genes showing the same expression pattern in CCRI12 and CCRI24, most differentially expressed *ARF* and *GH3* genes (70.5% of *ARF*s, 60.9% of *GH3*s) showed a comparable degree of expression change between AN_vs_AE and BN_vs_BE, of about half of the differentially expressed *Aux/IAA* and *SAUR* genes (29/56 (51.8%) *ARF*s, 26/52 (50.0%) *GH3*s) showed a different degree of expression change (the difference of log_2_FC greater than 1) between AN_vs_AE and BN_vs_BE (Figure 4). For example, the homologous *ARF* genes *Ghir_A10G002560* and *Ghir_D10G003340* showed a significant downregulation in CCRI24 during the transformation from NEC to EC, while no significant expression change was found in CCRI12 (Figure 5a). Similar cases were found in the *Aux/IAA* gene *Ghir_A05G002590* and *SAUR* gene *Ghir_A03G022510* (Figure 5b,d). Moreover, for the *Aux/IAA* genes *Ghir_A09G024180*, *Ghir_D05G004880*, and *Ghir_A06G020380*; the *GH3* genes *Ghir_A01G008830* and *Ghir_A05G040190*; and *SAUR* gene *Ghir_D13G010120*, they were upregulated or downregulated with a distinct higher ratio in CCRI24 than in CCRI12 (Figure 5).

**Figure 6:**
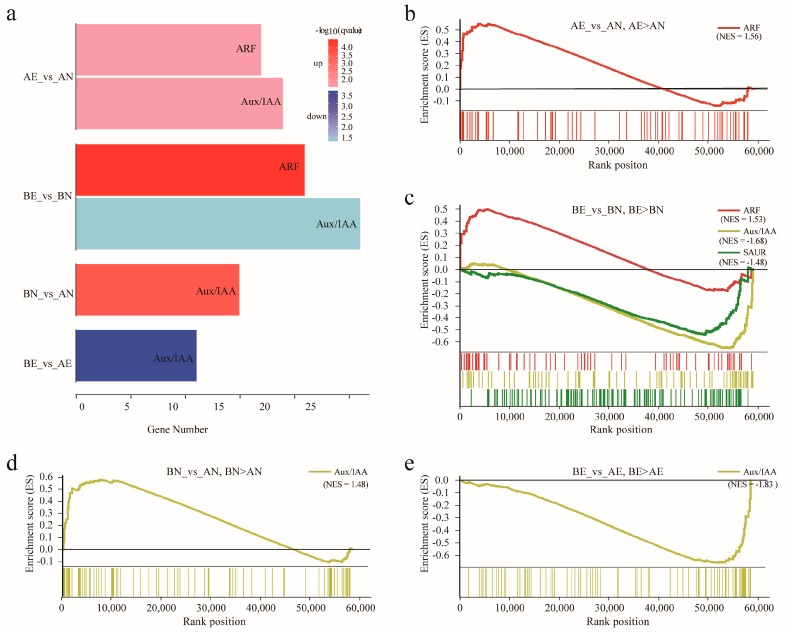
Enrichment results of *ARF*, *Aux/IAA*, *GH3*, and *SAUR* genes during the acquisition of embryogenic potential. (**a**) Over-representation analysis plots of the hypergeometric test based on differentially expressed *ARF*, *Aux/IAA*, *GH3*, and *SAUR* genes. Enriched auxin early response gene families are shown by the colored bars, enriched families in the upregulated and downregulated DEGs are colored by red and blue, respectively. The different shade of the red or blue color indicates the different degree of enrichment represented by the minus of log 10 transformed q values. (**b–e**) GSEA plots of *ARF*, *Aux/IAA*, *GH3*, and *SAUR* family gene sets in different comparison pairs, significant enriched family gene sets are shown in the enrichment score (ES) plots by different colors. Normalized enrichment scores (NES) for each enriched family set is shown in brackets. A positive NES value indicates that the family gene set was positively associated with the indicated relativeness in the comparison pair. A negative NES value indicates that the family gene set was negatively associated with the indicated relativeness in the comparison pair.

Over-representation analysis of the hypergeometric test based on all (in both AN_vs_AE, BN_vs_BE, AN_vs_BN, AE_vs_BE comparison pairs) the differentially expressed genes identified in transcriptomic sequencing found that the *ARF* and *Aux/IAA* families were significantly over-represented in both CCRI12 and CCRI24 during embryogenic potential acquisition of the callus. Both *ARF* and *Aux/IAA* families were over-represented in the upregulated DEGs identified in AN_vs_AE, while in BN_vs_BE, only the *ARF* family was over-represented in upregulated DEGs as the *Aux/IAA* family was over-represented in downregulated DEGs. In comparison, the AN_vs_BN and *Aux/IAA* family was enriched in upregulated DEGs (Figure 6a).

Gene set enrichment analysis was conducted based on all genomic gene expression profiles, with the *ARF*, *AuxIAA*, *GH3*, and *SAUR* families defined as gene sets, respectively. Results showed that in CCRI12, the *ARF* family gene set was significantly upregulated in AE when compared with AN using the nominal *p*-value < 0.05 and FDR *q*-value < 0.05 as criteria. In CCRI24, the *ARF* family gene set was upregulated in BE when compared with BN, and the *Aux/IAA* and *SAUR* family gene sets were significantly upregulated in BN when compared with BE. When comparing AN versus BN, the *Aux/IAA* family gene set was significantly upregulated in BN when compared with AN (Figure 6b–e). These results were almost consistent with the above over-representation analysis.

### 3.5. Co-Expression of ARF, Aux/IAA, GH3, and SAUR Genes and Somatic Embryogenesis Related Genes during Cotton Somatic Embryogenesis

Co-expression analysis was conducted among the *ARF*, *Aux/IAA*, *GH3*, and *SAUR* families. The result showed that the majority of DEGs in all of the four auxin early response families showed a significant expression correlation with each other both within intra- and inter-families (Table 3, Supplementary Figure S6), suggesting that the *ARF*, *Aux/IAA*, *GH3*, and *SAUR* families are closely collaborated in auxin signaling regulation during upland cotton somatic embryogenesis.

**Figure 7:**
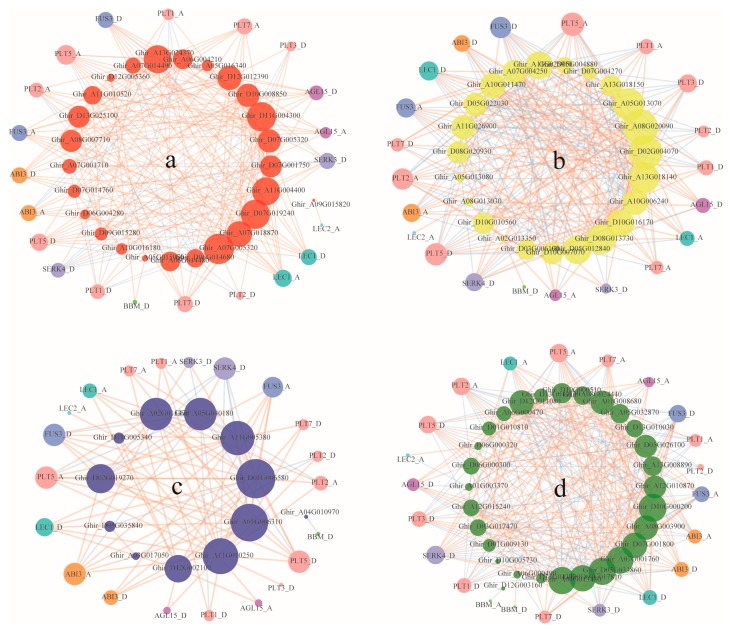
Co-expression networks of somatic embryogenesis related genes and four auxin early response gene families *ARF* (**a**), *Aux/IAA* (**b**), *GH3* (**c**), and *SAUR* (**d**). Reported somatic embryogenesis related genes *ABI3*, *AGL15*, *FUS3*, *LEC1*, *LEC2*, *PLTs*, *BBM*, and *SERKs* were identified in *G. hirsutum* and are represented by circle nodes in different colors. The red line links two nodes, indicating a positive correlation between these two genes, while the blue line indicates a negative correlation between genes. Only the intercorrelationship between auxin early response genes and somatic embryogenesis related genes and the intracorrelationship in auxin early response genes are shown. The size of nodes represents the degree of co-expression relations.

Somatic embryogenesis is a complex developmental process involved in the regulation of many genes and is influenced by various external environment factors including plant hormones. Up to now, a number of genes have been identified to play roles in somatic embryogenesis such as *LEC1*, *LEC2*, *ABI3*, *AGL15*, *FUS3*, *PLT*s, *BABY BOOM* (*BBM*), *WUSCHEL* (*WUS*), *SERK*s, and so on [53,54,55,56,57,58,59,60]. To investigate the co-expression relation between auxin early response genes and reported somatic embryogenesis related genes, we constructed co-expression networks of these genes based on their expression profiles during somatic embryogenesis. A total of 26 *ARF*, 23 *Aux/IAA*, 12 *GH3*, and 29 *SAUR* genes were tightly correlated with somatic embryogenesis related genes that had Pearson’s correlation coefficients > 0.98 and a p-value < 0.05 (Table 4, Figure 7). For *ARF*, *Aux/IAA*, *GH3*, and *SAUR* families, about half of these co-expressed auxin early responsive genes were greatly correlated with somatic embryogenesis related to *ABI3*, *FUS3*, *PLT2*, and *PLT5* genes (except that 30.8% of co-expressed *ARF* genes were correlated with *PLT2*). About 40% of co-expressed auxin early responsive genes were correlated with *LEC1* and *SERK4*, except for *ARF* family where only 30.8% co-expressed genes were correlated with *SERK4*. More than 30% of co-expressed *ARF*, *Aux/IAA*, and *SAUR* genes were correlated with *PLT1* and *PLT7*, as the counterpart percentage in the *GH3* family was 25%, 50%, and 25%, respectively. Over 20% of co-expressed auxin early responsive genes were correlated with *AGL15* and *SERK3* for the *ARF*, *Aux/IAA*, *GH3*, and *SAUR* families. About 40% of co-expressed *Aux/IAA* and *SAUR* genes were correlated with *PLT3*, as only 19.2% and 8.3% in the *ARF* and *GH3* families, respectively. Few auxin early responsive genes were correlated with *BBM* and *LEC2*, however, no gene was correlated with *WUS* (Table 4).

We constructed four networks to show the co-expression relations between somatic embryogenesis related genes and the auxin early response gene families *ARF*, *Aux/IAA*, *GH3*, and *SAUR*, respectively (Figure 7). Results showed that each auxin early response gene may co-express with multiple different somatic embryogenesis related genes, and vice versa, each somatic embryogenesis related gene may co-express with different auxin early response genes. The majority of co-expressed relations between somatic embryogenesis related genes and the *ARF* and *GH3* family genes were positive, while the *Aux/IAA* and *SAUR* families had a greater negative relation with somatic embryogenesis related genes (Figure 7).

In the *ARF* family, genes co-expressed with most somatic embryogenesis related genes were positively correlated with some exceptions. *SERK4_D* and *PLT1_A* were principally co-expressed with the *ARF* genes negatively. Half of the *PLT1_D* and *PLT2_D* were co-expressed and a few *ABI3_A*, *FUS3_D*, *PLT2_A*, *PLT5*, *PLT3_D*, and *SERK3_D* co-expressed *ARF* genes were negatively correlated with these somatic embryogenesis related genes (Figure 7a). Similar results were found in the *GH3* family, as most somatic embryogenesis related genes were positively correlated with *GH3* genes, but *SERK4_D* was negatively correlated with *GH3* genes (except for *Ghir_A05G040180*). Furthermore, *ABI3_A*, *FUS3_D*, *PLT1_A*, *PLT1_D*, *PLT2_A*, *PLT2_D*, *PLT5_A*, *PLT5_D*, and *SERK3_D* sometimes showed a negative correlation with some of the co-expressed *GH3* genes (Figure 7c).

In the *Aux/IAA* and *SAUR* families, genes co-expressed with somatic embryogenesis related genes in complex ways: somatic embryogenesis related genes positively and negatively correlated with *Aux/IAA* or *SAUR* family genes at the same times (Figure 7b,d). Nevertheless, we found that *PLT2_D* was principally negatively correlated with *Aux/IAA* genes and absolutely negatively correlated with *SAUR* genes, while *PLT1_A* was principally negatively correlated with *SAUR* genes.

## 4. Discussion

Auxin early response genes that play a key component in auxin homeostasis maintenance and signaling have been widely characterized in many plants. There were 23 ARFs, 29 Aux/IAAs, 20 GH3s, and 79 SAURs identified in *Arabidopsis* [17,22,61,62,63]; 17 ARFs, 26 Aux/IAAs, 15 GH3s, and 99 SAURs in tomato [64,65,66,67]; 22 ARFs, 26 Aux/IAAs, 11 GH3s, and 70 SAURs in citrus [68]; 16 ARFs, 27 Aux/IAAs, 10 GH3s, and 61 SAURs in cucumber [69]; 25 ARFs, 31 Aux/IAAs, 12 GH3s, and 58 SAURs in rice [70,71,72,73]; and 35 ARFs, 31 Aux/IAAs, 13 GH3s, and 79 SAURs in maize [74,75,76,77]. In the present study, 71 ARFs, 86 Aux/IAAs, 63 GH3s, and 194 SAURs were identified in upland cotton. All four auxin early response gene families had more gene members in upland cotton than many other plants, which may be largely attributed to the genome duplication of the allotetraploid upland cotton, thought to be derived from the allopolyploidization of A-subgenome and D-subgenome, in line with the result that segmental duplications widely exist in the upland cotton *ARF* family. Half the number of upland cotton *ARF* genes was nearly equal to that of maize (~2.3 G), which has a similar size of genome with upland cotton (~2.5 G), but about 1.5 fold of that in *Arabidopsis*, suggesting that the *ARF* family in upland cotton was relatively conserved when compared to maize, but somewhat expanded when compared to *Arabidopsis*. The number of upland cotton *Aux/IAA* genes was about 2.8- and 3-folds of that in maize and *Arabidopsis*, respectively, implying the obvious expansion of the *Aux/IAA* family in upland cotton. Only two genes were found to be involved in tandem duplication, while segmental duplication was extensively occurred. Therefore, genome duplication and segmental duplication played important roles in the expansion of *Aux/IAA* in upland cotton. The *GH3* and *SAUR* families were largely expanded in upland cotton when compared to many other plants. For the *GH3* family, there were 13 *GH3* genes derived from tandem duplication in upland cotton, which may contribute to the expansion of the *GH3* family in some degree. Nevertheless, when these tandem duplicates were excluded, the number of remaining *GH3* gene members in upland cotton was still higher than twice of that in *Arabidopsis*. Collinearity analysis revealed that segmental duplication also extensively contributed to *GH3* family expansion. Further collinearity investigation found that the two *GH3* gene members, *Ghir_A03G020320* and *Ghir_D02G021740*, were subjected to extensive segmental duplication as both genes had multiple corresponding syntenic genes on different chromosomes (chromosome 5 and 4, respectively). Phylogeny revealed that these two genes were orthologous genes of *AtGH3.1* (*AT2G14960*), which encoded a protein similar to IAA-amino synthases [22]. However, transcription profiles showed that only half of the two *AtGH3.1*-like genes and their homologs were actively expressed during the embryogenic potency acquisition of cotton callus. This implies the functional divergence of these gene duplicates during evolution. Aside from genome duplication, the *SAUR* family expansion can be largely attributed by the tandem duplication in upland cotton, where each gene is more likely to repeat many times (Figure 2a).

As tandem duplication extensively existed in the two greatly expanded *GH3* and *SAUR* families, the involved genes were further investigated. The majority of tandem duplicates derived from *GH3* genes were distributed in Group II, which was likely to be upland cotton specific that contained no *Arabidopsis* gene (Figure 1c), implying that the expansion of the *GH3* family in upland cotton may result from the divergent evolution of *Arabidopsis* and upland cotton, largely bringing new specific functions for upland cotton. Other *GH3* tandem duplicates involving eight genes belonged to Groups IV and V. The four *GH3* tandem duplicates in Group IV were orthologous genes of *AtGH3.11* (*AT2G46370*), also named *AtJAR1*, which acts as a positive regulator of JA signaling by encoding a jasmonate-amino synthetase that catalyzes jasmonyl-isoleucine (JA-Ile) conjugation, then promotes the interaction between JAZ1 repressor and COI1 [78,79]. A functional study revealed that *AtJAR1* was involved in pathogen defense and wound responses. Thus, the duplication of *AtJAR1* homologous genes in upland cotton may enhance its JA signaling and confer resistance to diverse biotic and abiotic stresses. The four *GH3* tandem duplicates in Group V were orthologous genes of *AtGH3.5* and *AtGH3.6*, encoded IAA-amino synthases, and participated in auxin homeostasis maintenance. Almost all tandem duplicated *SAUR* genes in upland cotton were distributed in Groups III, V, and VII. Tandem duplicates in Group III were orthologous genes of *AtSAUR1*–*AtSAUR5* with unknown function. Transcription profiles showed that only some of them were actively expressed during the embryogenic potency acquisition of cotton callus, implying that these duplicates may function in different developmental and physiological processes. Tandem duplicates in Group V and VII were orthologous genes of *AtSAUR61*–*AtSAUR68* and AtSAUR48, respectively; however, all these tandem duplicated genes showed a low expression level during the embryogenic potency acquisition of cotton callus, so we speculated that they may function in other physiological processes.

To investigate the roles of auxin early response genes in upland cotton somatic embryogenesis, we profiled their expression patterns during embryogenic potency acquisition of the callus. The *ARF* and *Aux/IAA* family genes were extensively expressed in this process, while only a small part of the *GH3* and *SAUR* family genes was active. Furthermore, more than 60% of the *ARF* and *Aux/IAA* family genes showed differential expression during the process from NEC to EC, suggesting that the *ARF* and *Aux/IAA* family play crucial roles in the regulation of auxin-mediated somatic embryogenesis in upland cotton. Though only the minority of *GH3* and *SAUR* genes showed active expression in embryogenic potency acquisition, the majority of these active genes were differentially expressed in NEC compared to EC, implying their regulatory roles in upland cotton somatic embryogenesis. When compared, the expression profiles of the auxin early response genes between cultivars with different regeneration abilities, the *ARF* and *GH3* genes were more likely to exhibit comparable expression patterns, while about half of the *Aux/IAA* and *SAUR* genes did not. Typically, during the transformation from NEC to EC, the *Aux/IAA* gene *Ghir_A05G002590* and the *SAUR* gene *Ghir_A03G022510* were almost specifically expressed in the NEC of CCRI24, and downregulated significantly into low expression levels in CCRI24, while maintaining low expression levels throughout the transformation in CCRI12 (Figure 5). The high accumulation of transcripts of these two genes in the NEC may be conducive to initiating the embryogenic transformation into EC. Subsequent significant decreased expression of these genes may be tightly regulated by the sharply increasing IAA content in the callus. Additionally, it is noteworthy that the *Aux/IAA* gene *Ghir_D05G002730* exhibited obvious opposite expressed trends between CCRI12 and CCRI24 during callus embryogenic potency acquisition as significantly upregulated in CCRI12, but downregulated in CCRI24 (Figure 5). The different expression patterns of this gene between CCRI12 and CCRI24 may be associated with the difference of their somatic embryogenesis abilities. Further expression profiles analysis found that this gene had a significantly higher expression level in the NEC of CCRI24 than that of CCRI12, just like its homologous gene *Ghir_A05G002590* as described above. Therefore, we speculated that these genes may be negatively correlated with the transformation from NEC to EC, but be of benefit for triggering embryogenic transformation at high expression level. Overexpression of these genes in NEC may help improve the efficiency of somatic embryogenesis in reluctant cotton lines. Based on the results of enrichment analysis, the *ARF* family was enriched in upregulated genes during callus embryogenic potency acquisition in both CCRI12 and CCRI24 (Figure 6), suggesting the general positive regulatory roles of the *ARF* family in the embryogenic potency acquisition of upland cotton callus. The *Aux/IAA* family was generally downregulated during callus embryogenic potency acquisition in CCRI24, while enriched in downregulated genes in CCRI12. At the same time, the *Aux/IAA* family was generally significantly upregulated in the NEC of CCRI24 when compared to that of CCRI12 (Figure 6). This suggests that the overall high expression level of the *Aux/IAA* family genes or some critical gene members in NEC may be a prerequisite for the initiation of the embryogenic transformation into EC, even though there was subsequent downregulation during the transformation, as above-mentioned. Furthermore, the *SAUR* family, as effectors of hormone signaling [25], was enriched in CCRI24, but not in CCRI12 during transformation from NEC to EC, indicating the more effective regulation of auxin signaling in CCRI24.

Auxin early response genes in the *ARF*, *Aux/IAA*, and *SAUR* family were tightly co-expressed during the embryogenic potency acquisition of upland cotton callus, suggesting the close coordination of these genes in auxin signaling mediated somatic embryogenic transformation. About half of the differentially expressed *ARF*, *Aux/IAA*, *GH3*, and *SAUR* genes were co-expressed with previously reported somatic embryogenesis related genes, implying the associated regulation between them. The *ARF* and *GH3* family genes are likely to show a synergistic effect with somatic embryogenesis related genes as there is a generally positive correlation between them. Auxin early response genes were found co-expressed with most of the known somatic embryogenesis related genes including the *ABI3*, *FUS3*, *LEC1*, *PLT*s (*PLT1*, *PLT2*, *PLT3*, *PLT5*, *PLT7*), *AGL15*, and *SERK*s (*SERK3* and *SERK4*) genes. We speculate that these somatic embryogenesis related genes are implicated in upland cotton somatic embryogenesis by regulating or responding to auxin early response genes, or are mediated by *ARF*, *Aux/IAA*, *GH3*, and *SAUR* gene-involved auxin signaling up to some extent. While no auxin early response gene was co-expressed with *WUS*, it was supposed that *WUS* may regulate upland cotton somatic embryogenesis through other pathways, or directly regulated target genes downstream of the auxin signaling. In the present study, auxin early response genes were revealed to be co-expressed with a number of *PLT* genes during the embryogenic potency acquisition of upland cotton callus. Different *PLT* gene members showed a different co-expression pattern with auxin early response, where there were obviously more auxin early response genes co-expressed with *PLT2* and *PLT5* than *PLT1* and *PLT7*, while only a few auxin early response genes were co-expressed with *PLT4*/*BBM*. Furthermore, *PLT3* is more likely to be co-expressed with *Aux/IAA* and *SAUR* genes than *ARF* and *Aux/IAA* genes. These results suggest the complex regulatory patterns of *PLT* genes in somatic embryogenesis. Even though there is functional overlap and redundancy of *PLT* genes in plant development, each *PLT* gene member had specific co-regulation or response patterns during somatic embryogenesis. Previous studies have identified some *PLT* genes (*PLT1* and *PLT2*) as late auxin response genes and necessary for zygotic embryo development [80,81]. *PLT2* and *PLT4*/*BBM* induced somatic embryogenesis mediated by auxin signaling [56,82], while the functional and molecular mechanism of *PLT* genes in somatic embryogenesis remain unclear. The co-expression findings in our study provide valuable information for the exploration of the functional mechanism of *PLT*s in somatic embryogenesis. 

## 5. Conclusions

In the present study, we conducted a genome-wide identification and comprehensive phylogenetic analysis of auxin early response genes in upland cotton including *ARF*, *Aux/IAA*, *GH3*, and *SAUR* family members, which act as a key component of auxin signaling. The transcriptional dynamics of these auxin early response genes during embryogenic competence acquisition of cotton callus were further focused, based on transcriptomic analysis. The results revealed that auxin early response genes were extensively involved and played important roles in the embryogenic transformation of cotton callus. The expression level difference of the *Aux/IAA* family genes between different cultivars may be associated with their difference in somatic embryogenesis capacity. Co-expression analysis implied that most previously reported somatic embryogenesis related genes were implicated in plant somatic embryogenesis by interacting with auxin early response genes.

## Figures and Tables

**Table 1:** Characteristics of identified auxin response factor (ARF), Auxin/Indole-3-Acetic Acid (Aux/IAA), Gretchen Hagen3 (GH3), and small auxin upregulated RNA (SAUR) genes in upland cotton.

Gene Family	Domains/Motifs	Number of Genes
ARF		71
	DBD, ARF-AD ^a^, Aux/IAA	24
	DBD, ARF-RD ^a^, Aux/IAA	47
Aux/IAA		86
	Domains I, II, III, IV	67
	Domains I, II, III	1
	Domains II, III, IV	13
	Domains I, II	1
	Domains I, IV	1
	Domains II, III	1
	Domains III, IV	2
GH3		63
	GH3	47
	2 × GH3	8
	GH3, JAR1	8
SAUR		194
	SAUR, Motifs 1 ^b^, 2, 3	167
	SAUR, Motifs 1, 2	9
	SAUR, Motifs 1, 3	2
	SAUR, Motifs 2, 3	12
	SAUR, Motif 2	3
	SAUR, Motif 3	1

^a^ AD means activator domain, RD means repressor domain. ^b^ Motif 1 is auxin-inducible.

**Table 2:** Statistics of differentially expressed *ARF*, *Aux/IAA*, *GH3*, and *SAUR* genes during transformation from NEC to EC in upland cotton somatic embryogenesis. Differentially expressed genes (DEGs) were identified in four comparison pairs, respectively.

DEGs		AN_vs_AE	BN_vs_BE	AN_vs_BN	AE_vs_BE	Total
ARF		32	38	9	2	44
	up	17	21	5	1	-
	down	15	17	4	1	-
Aux/IAA		35	38	17	11	56
	up	19	12	15	0	-
	down	16	26	2	11	-
GH3		14	17	4	1	23
	up	9	8	2	0	-
	down	5	9	2	1	-
SAUR		36	43	7	4	52
	up	15	10	6	0	-
	down	21	33	1	4	-

**Table 3:** Statistics of co-expressed genes among auxin early response families.

Auxin Early Response Family	DEGs	Number (Percentage) of DEGs Co-Expressed with			
		ARF	Aux/IAA	GH3	SAUR
ARF	44	38 (86.4%)	39 (88.6%)	37 (84.1%)	40 (90.9%)
Aux/IAA	56	39 (69.6%)	41 (73.2%)	39 (69.6%)	41 (73.2%)
GH3	23	18 (78.3%)	19 (82.6%)	17 (73.9%)	20 (87.0%)
SAUR	52	48 (92.3%)	49 (94.2%)	45 (86.5%)	49 (94.2%)

**Table 4:** Statistics of the numbers of auxin early response genes co-expressed with somatic embryogenesis related genes.

SE Related Genes	Number (Percentage) of Co-Expressed Auxin Early Response Genes			
	ARF	AuxIAA	GH3	SAUR
ABI3	14 (53.8%)	15 (65.2%)	8 (66.7%)	18 (62.1%)
AGL15	8 (30.8%)	8 (34.8%)	3 (25.0%)	9 (31.0%)
FUS3	12 (46.2%)	13 (56.5%)	8 (66.7%)	16 (55.2%)
LEC1	10 (38.5%)	11 (47.8%)	5 (41.7%)	12 (41.4%)
LEC2	1 (3.8%)	1 (4.3%)	1 (8.3%)	2 (6.9%)
PLT1	8 (30.8%)	8 (34.85)	3 (25.0%)	11 (37.9%)
PLT2	8 (30.8%)	14 (60.9%)	6 (50.0%)	14 (48.3%)
PLT3	5 (19.2%)	10 (43.5%)	1 (8.3%)	12 (41.4%)
PLT7	10 (38.5%)	7 (30.4%)	3 (25.0%)	9 (31.0%)
BBM	2 (7.7%)	1 (4.3%)	1 (8.3%)	2 (6.9%)
PLT5	12 (46.2%)	17 (73.9%)	7 (58.3%)	16 (55.2%)
WUS	0 (0.0%)	0 (0.0%)	0 (0.0%)	0 (0.0%)
SERK3	9 (34.6%)	5 (21.7%)	3 (25.0%)	9 (31.0%)
SERK4	8 (30.8%)	10 (43.5%)	7 (58.3%)	15 (51.7)
Total	26	23	12	29

## Supplementary Materials

The following are available online at https://www.mdpi.com/2073-4425/10/10/730/s1, Figure S1. Multiple sequence alignment of ARF proteins from *Arabidopsis* and upland cotton showed conserved residues or motifs. Figure S2. Multiple sequence alignment of Aux/IAA proteins from *Arabidopsis* and upland cotton showed conserved residues or motifs. Figure S3. Multiple sequence alignment of GH3 proteins from *Arabidopsis* and upland cotton showed conserved residues or motifs. Figure S4. Multiple sequence alignment of SAUR proteins from *Arabidopsis* and upland cotton showed conserved residues or motifs. Figure S5. Domain and exon–intron organization structure of identified *ARF* (**a**), *Aux/IAA* (**b**), *GH3* (**c**), *SAUR* (**d**) family gene members in *Arabidopsis* and upland cotton. Figure S6. Co-expression network showing the tight correlationship among the four auxin early response families. Table S1. The summary of expression of auxin early response genes during the transformation from NEC to EC. Table S2. Primers used for qRT-PCR validation of expression pattern of auxin early response genes.
