# Supplementary material for: Genome-Wide Analysis of Cotton Auxin Early Response Gene Families and Their Roles in Somatic Embryogenesis
Source: Genes (Basel). 2019 Sep 20;10(10):730. doi: 10.3390/genes10100730 (PMC6827057; doi:10.3390/genes10100730)
Supplement: Supplementary file 1 [file genes-10-00730-s001.zip › Supplementary Table 1-2.pdf]

Table S1. The summary of expression of auxin early response genes during the transformation from NEC to EC.

Families	Number of expressed genes during embryogenic transformation						Total
	TPM<1	1<TPM<5	5<TPM<10	10<TPM<30	30<TPM<50	TPM>50	
ARF	4	3	14	0	40	10	71
Aux/IAA	14	15	5	0	31	21	86
GH3	26	10	9	0	15	3	63
SAUR	114	45	35	0	0	0	194

Table S2. Primers used for qRT-PCR validation of expression pattern of auxin early response genes.

Families	Gene ID	Forward primer	Reverse primer
ARF			
	Ghir_A10G002560	AGAACTTTTGCAGATGCGGTC	TTACAGACCGGAACTATGAACTCC
	Ghir_D10G003340	CACTTTTGCAGATGCGGTCC	TACAGACCGGAACTATGAACTCC
Aux/IAA			
	Ghir_A05G002590	TGGGAATATTGCTCCAGCTTCA	AGGGTGAGAAGCCATCGTATTC
	Ghir_A06G020380	TGCTTGTCGGTGATGTCCC	TGCTTCCAAGGTTGATAACGG
	Ghir_A09G024180	AGTTTGGCTTCAGCACTCGATA	CCATCCAAAAGCTTAGATGGCA
	Ghir_D05G002730	TGGGAATATTGCTCCAGCTTCA	TCATTCTTCGGAGGGTGAGC
	Ghir_D05G004880	GCCTCCACTAACATTACCTCCA	GCTTGGACAAGCTGTTGCTC
	Ghir_D09G023320	GATCAATCTGTCCCCCATGACG	AGCTGTTCATCCTGTAAGACCT
GH3			
	Ghir_A01G008830	GCCCTGTTAACTGCTGTGATGA	GCCACAAGGAGTTTCAGTCTCT
	Ghir_A05G040190	TAAACCCGTTACAGGGCACTT	AGTTTCAATCTCTGGTCTGGCA
SAUR			
	Ghir_A03G022510	ATTTATGGACACCTCACGTTGC	AGACATAATCAATGCTTTCTCCACT
	Ghir_D13G010120	AAGAGTTCGGCTTCGATCACC	TCGTTGAAGAGGACAAGTATGC
