# Supplementary material for: Genome-Wide Analysis of Cotton Auxin Early Response Gene Families and Their Roles in Somatic Embryogenesis
Source: Genes (Basel). 2019 Sep 20;10(10):730. doi: 10.3390/genes10100730 (PMC6827057; doi:10.3390/genes10100730)

Supplementary Figure 5. Domain and exon-intron organization structure of identified ARF (a), Aux/IAA (b), GH3 (c), SAUR (d) family gene members in *Arabidopsis* and upland cotton. Conserved functional domains coding regions were marked on exons by different colored rectangles.

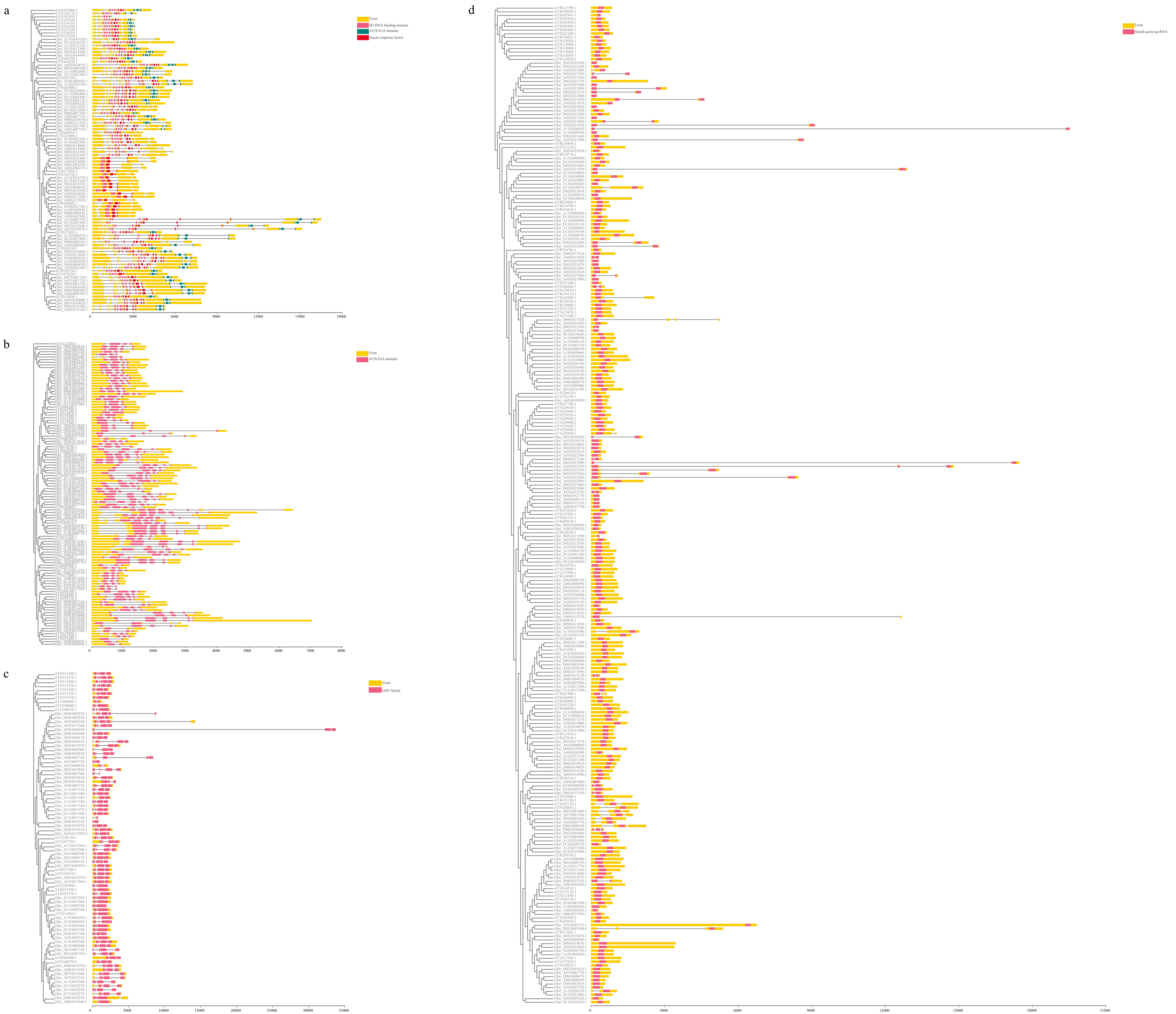

Supplement: Supplementary file 1 [file genes-10-00730-s001.zip › Supplementary Figure 5.pdf]
