# Supplementary material for: Genome-Wide Analysis of Cotton Auxin Early Response Gene Families and Their Roles in Somatic Embryogenesis
Source: Genes (Basel). 2019 Sep 20;10(10):730. doi: 10.3390/genes10100730 (PMC6827057; doi:10.3390/genes10100730)

Supplementary Figure 6. Co-expression network showing the tight correlationship among the four auxin early response families in upland cotton. *ARF*, *Aux/IAA*, *GH3*, *SAUR* genes were respectively represented by red, yellow, blue, green circle nodes.

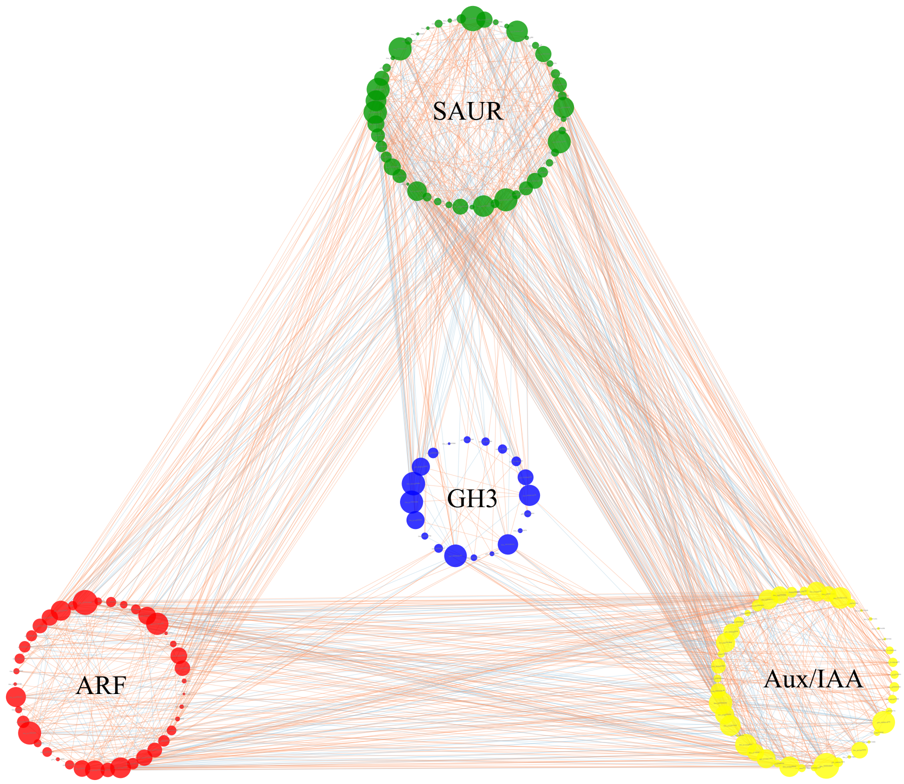

Supplement: Supplementary file 1 [file genes-10-00730-s001.zip › Supplementary Figure 6.pdf]
