# Supplementary material for: Genome-Wide Analysis of Cotton Auxin Early Response Gene Families and Their Roles in Somatic Embryogenesis
Source: Genes (Basel). 2019 Sep 20;10(10):730. doi: 10.3390/genes10100730 (PMC6827057; doi:10.3390/genes10100730)

Supplementary Figure 3. Multiple sequence alignment of GH3 proteins from *Arabidopsis* and upland cotton showed conserved residues or motifs.

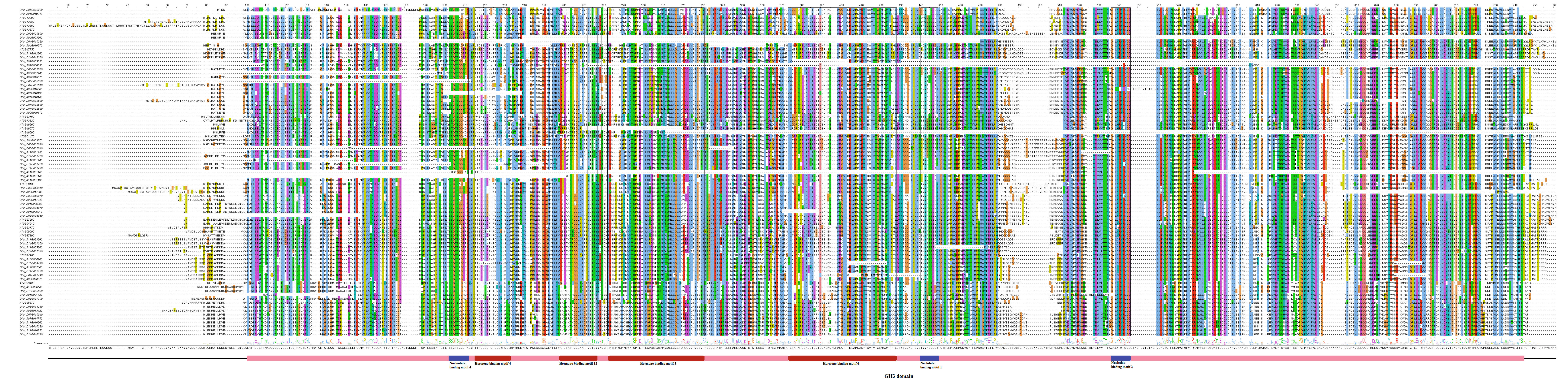

Supplement: Supplementary file 1 [file Supplementary Figure 3.pdf › Supplementary Figure 3.pdf]
